# Anaphoric Strategies Across Language Modalities: A Comparison Between Catalan and Catalan Sign Language (LSC)

**DOI:** 10.1007/s10936-017-9540-9

**Published:** 2017-11-22

**Authors:** Laia Mayol, Gemma Barberà

**Affiliations:** 1 Universitat Pompeu Fabra, Barcelona, Spain; 2 Université Paris 8 / CNRS, Paris, France

**Keywords:** Anaphora, Catalan, Catalan Sign Language (LSC), Overt pronouns

## Abstract

The goal of this paper is to compare the different anaphoric strategies that Catalan and Catalan Sign Language (LSC) use by means of a parallel corpus. In particular, our comparison is focused in an examination of the uses of overt subject pronouns in Catalan and how these uses are rendered in a language that exploits the visual-manual modality, such as LSC. As far as we know, this is one of the first studies to compare reference-tracking devices in a spoken and a signed language by means of a parallel corpus and incorporating both a descriptive and a theoretical perspective. All instances of overt pronouns in Catalan were analyzed and most of the data can be accounted with three factors: topic change, focus and contrast. As for LSC, the use of pronouns is rare and only few instances were found. Instead, other anaphoric strategies are used: while topic change and focus are primarily encoded with bare nouns, the expression of contrast relies on modality-specific features.

## Introduction

Natural languages are externalised in two different modalities. On the one hand, the auditory-oral modality is produced by the vocal tract and perceived by the auditory channel; on the other, the visual-spatial modality is produced with the hands and the upper body and perceived visually. Linguistic research has proven that, regardless of their inherent modality, spoken and signed languages share basic linguistic properties on the different grammatical levels (Sandler and Lillo-Martin 2006). Still, modality plays a role in shaping the expression of linguistic structure and in conveying meaning. This article presents and compares the different anaphoric strategies that spoken Catalan and Catalan Sign Language (*llengua de signes catalana, *LSC) use by means of a parallel corpus. In particular, our comparison is focused in an examination of the uses of overt subject pronouns in Catalan and how these uses are rendered in a language that exploits the visual-spatial modality, such as LSC.

Anaphora has been a topic of interest in linguistics and philosophy for a long time. Since anaphora resolution is a key aspect of language interpretation and the choice of an appropriate referring expression is a crucial aspect of language production, it is not surprising that many theories try to model these phenomena. In particular many efforts have been devoted to the challenging task of explaining the mechanisms that control pronoun choice and resolution. This task becomes doubly challenging for languages that have both overt and null pronouns. One of the goals of this paper is to present the factors that favour the use of overt pronouns in a null subject language like Catalan.

The other goal of the paper is to explore the anaphoric strategies present in LSC. Sign languages in general, and LSC in particular, use the three-dimensional space in front of the signer’s body (the so-called *signing space*) to establish coreferential relations. Signing space is used for articulatory reasons; that is, it is the area where the hands and the arms move, like the tongue is accommodated in the mouth in spoken language. But this is not the only function, since signing space also carries linguistic meaning. When a discourse referent is introduced, it is associated with a spatial area. This spatial area is used to further refer back to the discourse referent by means of a pointing sign directed to it, which may function as a pronoun or a determiner. Research into the mechanisms displayed in sign languages for reference-tracking has been so far rather limited, and it has partly concentrated on their acquisition. One of the few works is Morgan (2006), which establishes a simple hierarchy of referring expressions in terms of explicitness; that is of how much descriptive content they encode and, consequently, how transparent they are in the identification of its antecedent. Information structure in the visual-spatial modality is another domain where very few works are found. Wilbur (2012) and Kimmelman and Pfau (2016) present an overview of how information structure is conveyed in sign languages, and Kimmelman (2014) presents a contrastive analysis between Russian Sign Language and Sign Language of the Netherlands. Yet, detailed analyses of particular topics are still to be explored.

As far as we know, the present article is one of the first studies to compare reference-tracking devices in a spoken and a signed language by means of a parallel corpus incorporating both a descriptive and a theoretical perspective. The final aim is to show a correspondence in the classification of functions of anaphoric strategies that the two languages use, taking into account the different instantiations that serve a reference-tracking function. While spoken Catalan uses pronouns as the primary strategy for reference-tracking, LSC uses pronouns to a lesser degree and uses instead a rich array of anaphoric strategies. We have used two parallel corpora of the languages under study, which are based on the *Frog Stories*, a series of wordless pictures books. Several speakers and signers were asked to narrate these stories, which were presented to them only with illustration, and the narrations were recorded and transcribed. In the case of spoken Catalan, the Nocando corpus^1^ consists of a corpus of spoken narrative discourse (Brunetti et al. 2009) and seeks to establish a crosslinguistic taxonomy of noncanonical constructions. It contains three different stories, each one told by nineteen speakers. For the case of LSC, the data for the present study have been extracted from the LSC Corpus^2^ (Barberà et al. 2015). It contains data from 6 native signers (that is, signers with direct signing family and/or who attended specific schools), aged between 18 and 80 years. The conversations were all recorded in mixed couples (man and woman).

The article is structured as follows. In “Overt Pronouns in Catalan” section the three main factors triggering the presence of an overt subject in spoken Catalan are presented. “Anaphoric Strategies in Catalan Sign Language” section introduces the anaphoric strategies used in LSC that are relevant for the present purposes. In “Results” section these strategies are analysed according to each factor triggering overt marking as established in the present proposal. “Discussion and Conclusions” section discusses the main findings and concludes.

## Overt Pronouns in Catalan

Catalan, as other Romance languages like Italian or Spanish, is a null-subject language and has a double system of pronouns (Rigau 1986). In subject position, there is an alternation between overt pronouns (*ella* in 1a) and null pronouns (in 1b).^3^

**Table:** 

(1)	a.	**Ella** canta.
		She sing.3SG
		‘**She** sings.’
	b.	Canta.
		Sing.3SG
		‘Sings’

There are cases in which the overt pronoun is ungrammatical, as in 2a, cases in which it is optional, as in 2b, and cases in which it is mandatory, as in 2c (examples from Rigau 1989).

**Table:** 

(2)	a.	Has entrat i (* **tu**) has sortit.
		Have.2SG entered and **you** have.2SG left.
		‘You have entered and **you** have left.’
	b.	Quan (**ell**) va arrivar, tothom va escoltar.
		When he AUX.PST arrive, everyone AUX.PST listen
		‘When **he** arrived, everyone listened.’
	c.	En Pere és de Barcelona, però *(**tu**) ets de Girona.
		The Pere be.3SG from Barcelona, but you be.2SG from Girona
		‘Pere is from Barcelona, but **you** are from Girona.’

Our corpus consists in 71 instances of overt pronouns from the Nocando corpus. These 71 examples represent approximately 1.3% of the total number of subjects in the corpus (around 5500). Thus, the use of overt pronouns is quite restricted and clearly the default pronominal expression is the null pronoun. Still, the question remains of when the overt pronoun is used in a null-subject language. Many proposals address precisely this question. In this section we will review the three main factors, which can account for 87% of the data. These three factors are the expression of (i) topic change, (ii) focus, and (iii) contrast. We will also review some of the problematic cases in our data.

### Topic Change

It is a recurrent idea in the literature that while null pronouns mark topic continuation, overt pronouns mark topic change in some Romance languages. For instance, variationist studies that take into account several factors to account for overt pronouns systematically signal topic change (often called switch reference) as one of the most important factors, as in the work by Cameron (1992) and Silva-Corvalán (1977) for Spanish.

The hypothesis in Vallduví (1992) is that null and overt pronouns differ in fundamental ways regarding their contribution to information structure: while overt pronouns work towards constructing the information structure of a text, null pronouns do not. In his tripartite model, sentences are divided into focus and ground, where the ground is further divided into link and tail. Information packaging is seen as instructions for information update. The focus is the actual update potential of the sentence. In contrast, the ground indicates how the information update must take place. The link indicates where the focus should go (in which file, following File Change Semantics Heim 1982), and the tail how the information must be updated. All sentences have a focus, while both elements of the ground are optional. Linkless sentences occur when (i) no particular file is relevant (such as, presentational or existential sentences) and (ii) when there is a relevant file/topic, but it need not be mentioned, because it can be inferred from context. The second case includes those pairs of sentences in which a sentence S$_{n}$ shares its topic with S$_{n-1}$. In this situation, S$_{n}$ need not have a link, it may have a null pronoun. In contrast, the use of a link in two adjacent sentences will imply a change of locus of update from S$_{n-1 }$to S$_{n}$. This includes the use of an overt pronoun.

 Carminati (2002) proposes that the variation between overt and null pronouns is regulated by the Position of Antecedent Hypothesis (PAH). The PAH proposes that, within a sentence, null and overt pronouns have different antecedent biases: null pronouns prefer to retrieve an antecedent in the (highest) Spec IP, whereas overt pronouns prefer an antecedent in a lower syntactic position. This hypothesis is in accordance with Ariel (1990)’s proposal that more marked, informative forms tend to retrieve less salient antecedents, while unmarked, less informative forms tend to retrieve more salient antecedents.

Vallduví’s and Carminati’s ideas are similar, but not identical, since the former talks about topic change and the latter about subject change. Since subjects are often topics, both accounts will very often make the same predictions, but the topic change proposal can account for more cases than the subject change proposal.

This factor can account for 30% of the examples in our corpus (21 examples). (3) is such a case. In this example, the two main discourse referents are two frogs: a big frog and a small frog.^4^

**Table:** 

(3)	Altre cop tira la granoteta fora però, com que estan a l’aigua, cau a l’aigua i **ella** li fa llengotes.
	‘Again [null$_{\mathrm{bigfrog}}$] pushes that little frog outside, but since [null$_{\mathrm{bigfrog+littlefrog}}$] are in
	the water, [null$_{\mathrm{littlefrog}}$] falls in the water and **she** $_{\mathrm{bigfrog}}$ sticks the tongue out at her.’

At the beginning of the fragment, the speaker is talking about the big frog, which is the topic at that point. In the next clause, the subject of the verb ’to fall’ refers to the little frog and is realized through a null pronoun, which indicates that overt pronouns are not compulsory for a topic change if there is enough contextual bias. In the following clause, there is an overt pronoun, which switches the reference again (it refers back to the big frog). Since there is no contextual biasing in this example, the overt pronoun is needed to switch reference again. If there was a null pronoun instead, the hearer would interpret it as referring to the previous subject and not to the referent the speaker intended.

Of the overt pronouns used in a topic change situation, we can distinguish several special cases.

- Return to main topic: the pronoun may serve to close a segment and return to a previous main topic. For example, after an embedded clause (relative clause or clausal complement), which introduces a secondary topic, the pronoun may serve to go back to the main topic. In example (4), the boy is the main topic, which is temporarily overriden by a secondary topic (the boat), about which some information is added through two relative clauses. When the speaker wants to go back to the main topic, an overt pronoun is used to mark the switch.

**Table:** 

(4)	I el nen s’estava mirant un vaixell que deu ser com de paper que estava navegant
	per l’estany. I **ell** estava molt content mirant el vaixell.
	‘The boy is looking at the ship that is made of paper and that is sailing in the
	lake. And **he** was very happy looking at the ship.’

- Reference to a previous object: these are the cases that would be covered by the Position of Antecedent Hypothesis. The overt pronoun refers to the previous object and thus is used to change the topic of the sentence. (5) illustrates such a case:

**Table:** 

(5)	I la tortuga s’espanta molt fins que crida el xiquet. L’avisa i **ell** intenta buscar-la.
	‘The turtle gets scared and [null] calls the kid. [null] warns him and **he** tries to look for her.’

### Focus

There are 16 cases (23%) in our corpus in which the pronoun represents focal information. In these cases, there is no real choice between the two types of pronouns. Focal information is placed at the end of the main clause in Catalan, which is where the main pitch of the sentence is located. If the subject is focal information, the speaker cannot use a null pronoun: only a full pronoun can host the main pitch of the sentence in the sentence-final focal position. Otherwise, if a null pronoun were used, the main pitch would be placed on some other constituent and this would yield a different informational structure.

Apart from pragmatic considerations, there is independent evidence for considering these subjects as conveying focal information.

**Table:** 

1.	The pronoun appears in postverbal position.
(6)	La granota gran va dir “Aquesta no es queda aquí a casa meva, si hi sóc **jo**”.
	‘The big frog said “She will not stay here, in my place, if **I** am here”.’
2.	The pronoun appears in the focus position in a cleft or pseudo-cleft.
(7)	La mare s’enfada molt amb el nen perquè es pensa que ha sigut **ell** que l’ha enfonsat.
	‘The mother gets very angry with the child because she thinks that **he** was the one who sank it.’
3.	The pronoun appears together with a focal particle (*even, self, also *etc.).
(8)	A l’home li va caure el te, les ulleres, li va caure tot. Va caure fins i tot **ell** a terra.
	‘The man dropped the tea, the glasses, everything. Even **he** fell down.’

As mentioned, in none of these cases can a null pronoun be used. There is, however, a choice between using the pronoun or using a definite description. For example, in (7) above, the choice would be between the pronoun ‘ell’ and the noun phrase ‘el nen’ (‘the child’) (note that the same choice would be present in the English translation of the cleft).

## Contrast

It is well known that null-subject languages resort to overt pronouns in order to convey contrast. In our data, contrast accounted for 34% of the data (24 examples). We identified three different types of contrast: double contrast, implicit contrast and weak contrast (see also Mayol (2010) for more extensive discussion of the data and a review of the proposals about the contrastive import of pronouns). We should note that we don’t include here cases of contrastive focus (which would be covered in the previous subsection).

Let us start the discussion with double contrast. In these cases, we find a two-clause discourse in which the subject position of each clause is occupied by two different referents about which opposite events or states are predicated, as shown in (9).

**Table:** 

(9)	Ara **nosaltres** anirem a navegar per l’aigua i **tu** et quedaràs aquí sola.
	‘Now **we** will go mailing in the water and **you** will stay here on your own.’

In cases of double contrast, then, the alternatives being compared are explicit and, for each of the relevant discourse entity, it is conveyed whether they did (or did not do) whatever is predicated of them. This is different from what happens in instances of implicit contrast, in which the two contrasting alternatives are not explicit, but there is an implicit contrast between the antecedent of the pronoun and another entity, highly salient and identifiable in the context. This is what happens in (10): the second overt pronoun evokes an implicit contrast between the boy and the rest of the family and it is conveyed that the rest of the family, unlike the boy, was looking forward to the dinner.

**Table:** 

(10)	En el camí de tornada tots estan enfadats i ell, en canvi, està content perquè **ell** no
	tenia cap ganes d’anar-se’n a sopar.
	‘On the way home, they are all angry and he, in contrast, is happy, because **he**
	was not looking forward going out for dinner’.

Finally, there is a third type of contrast, weak contrast, in which it is conveyed that the speaker ignores or does not want to commit himself to whether the predicate is true of anyone else than the antecedent of the pronoun. This is illustrated in example (11): a waiter is asking a group of people what they want for dinner. The mother’s answer contains an overt subject pronoun, not because she is opposing her eating chicken to someone else not eating chicken, but rather because her answer is just a partial one: she has no information about what other people will eat.

**Table:** 

(11)	“Què voldran per sopar?” La mare diu: “Bé, doncs **jo** vull pollastre.” I el pare,
	“Doncs, **jo** vull sopa”.
	‘ “What will you have for dinner?” The mother says: “Well, **I**’ll have chicken”
	and the father says “Well, **I** will have soup”.’

In this paper we follow the proposal in Mayol (2010) according to which contrastive (non-focal) overt pronouns are Contrastive Topic markers, in the sense of Hara and van Rooij (2007). A Contrastive Topic triggers topic alternatives [see (12a)] and conveys an uncertainty contrast: an implicature that the truth of the alternatives is not known to the speaker [see (12b)].

**Table:** 

(12)	a.	Topic Alternatives:
		$\{\hbox {P}(\hbox {T}^{\prime }): \hbox {T}^{\prime } \, \in \, \hbox {Alt}(\hbox {T})\}$, where P is the property under discussion.
	b.	CT-Implicature (‘uncertainty contrast’):
		$\exists \hbox {T}' [\hbox {T}' \, \in \, \hbox {Alt}(\hbox {T})] [\lnot \hbox {K}_{\mathrm{sp} }(\hbox {P}(\hbox {T}^{\prime }))]$, where K $_{\mathrm{sp}}$ represents “the speaker knows that”.

This implicature is one of uncertainty; the speaker does not know whether the other alternatives are true or not. Thus, this implicature directly accounts for the weak contrast illustrated above. Furthermore, this uncertainty contrast can be coerced into a stronger exhaustive contrast, which conveys the implicature that the relevant topic alternative is false. This strengthening takes place if there are enough contextual cues: namely, there needs to be a salient relevant alternative either in the context [as in the implicit contrast cases such as (10)] or in the discourse [as in the double contrast cases such as (9)]. This exhaustive contrast is formalized in (13):

**Table:** 

(13)	Strenghtened CT-Implicature (‘exhaustive contrast’):
	$\exists \hbox {T}' [\hbox {T}' \in \hbox {Alt}(\hbox {T})] [K_{\mathrm{sp}}\lnot (\hbox {P}(\hbox {T}'))]$, where K $_{\mathrm{sp}}$ represents “the speaker knows that”.

### Unaccounted Examples

There are still some examples (around 13%) in the corpus data that are not covered by any of the previous factors. In these examples, the pronoun refers to the previous topic, it is not focal and there is no contrast, and still it is felicitous.

For example, consider (14)–(17). In all of them the pronoun is referring to the previous subject, it is not part of the focus and there is no contrast.

**Table:** 

(14)	En Pau que està molt content de tenir un altre animal de companyia, decideix que
	se’ls endurà a tots a fer una visita al parc. Abans, però [null] ja s’adona que la
	granota antiga, és a dir, la granota que **ell** ja tenia no sent una gran afecció cap a la nova.
	‘Pau, who is very happy of having another pet, decides that [null] will take them
	all to the park. However, before that, [null] realizes that the old frog, that is, the
	frog **he** already had does not like a lot the new frog.’
(15)	Aleshores quan [null] obre el regal s’adona que és una granota petita, però **ell** ja en tenia una.
	‘Then when [null] opens the present he realizes that there is a small frog, but **he** already had one.’
(16)	Tots estan força enfadats excepte en Pau que està content perquè ha pogut recuperar la seva granota, tot i que **ell** no s’havia adonat que s’havia escapat.
	‘They are all pretty angry except from Pau who is happy because [null] has been
	able to recover his frog, although **he** had not realized [null] had escaped.’
(17)	La senyora que no ha vist a la granota treu un biberó de la seva bossa i es disposa a donar-lo al nen mentres **ella** va llegint una revista.
	‘The lady who has not seen the frog takes a baby bottle from her bag and she is
	about to give it to the child while **she** is reading a magazine.’

The examples discussed in this section would still be felicitous without the overt pronoun and there would not be a significant change in meaning. A possible future line of research would be to investigate how pronouns can contribute in signalling and processing certain rhetorical relations in order to explain these unexpected uses. For instance, note that in (14)–(16), the overt pronoun is present in a segment that marks a rhetorical relation of violated expectation (Kehler 2002). One way to account for these examples would be to subsume them as cases of contrast. However, it is not immediately obvious how this could be formalized, since these examples do not fit neatly into any of the contrast types discussed above. Furthermore, even if this could be done, there would still be examples to account for, such as (17): no violated expectation is marked here and yet we find an overt pronoun to refer to a previous topic.

## Anaphoric Strategies in Catalan Sign Language

We turn now to Catalan Sign Language (LSC). LSC shows a rich array of strategies used in anaphoric contexts. In the *Frog Stories *the anaphoric strategies found in LSC may be divided in two main groups, namely fully specified referring expressions and underspecified ones. While the former may both introduce and refer back to a discourse referent already introduced, the latter are anaphorically dependent to the previously introduced antecedent. The categories included in each group are defined and exemplified below.

1. Fully specified referring expressions:
  (i) Noun phrases (NPs): Nouns followed or preceded by an index-handshape (fist closed and index finger extended) directed to signing space, which functions as the equivalent to determiners. In LSC, the prenominal or postnominal position of the determiner is irrelevant, as shown in (18).^5^

**Table:** 

(18)	a.	IX3 FROG
	b.	FROG IX3
		‘the frog’
  (ii) Bare nouns (BNs): Nouns used without any determiner or quantifier. Once the discourse referent has been introduced, a repetition by means of a BN triggers a definite reading.

**Table:** 

	____br
(19)	TREE BOY JUMP.
	‘The boy jumped into the tree.’
  2. Underspecified referring expressions:
  (i) Pronouns: Signs articulated with an index handshape and directed towards a spatial area in signing space, which substitute an entity already introduced. In some cases, LSC also uses thumb-handshape signs (fist closed and thumb extended), typical in contexts with non-present discourse referents.

**Table:** 

(20)	IX3 JUMP.
	‘He jumped.’
  (ii) Entity classifiers: Complex morphemes conveying movement and location information that function as an anaphoric device (Zwitserlood 2012). The classification of handshapes is established according to visual and geometrical properties of the antecedent. As underspecified referring forms, the antecedent needs to have been previously introduced by means of a full lexical sign (Fig. 1) in order for the classifier to get its meaning (Fig. 2).

**Table:** 

(21)	NIGHT FROG CL(8) “legged entity climbs up the window” ESCAPE
	CL(8) “legged entity jumps and walks away”.
	‘At night the frog climbed up the window and escape; it jumped and walked away.’

**Fig. 1:**
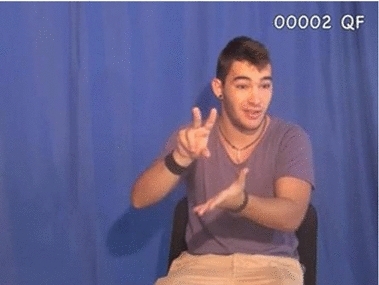
Lexical sign for ‘frog’

**Fig. 2:**
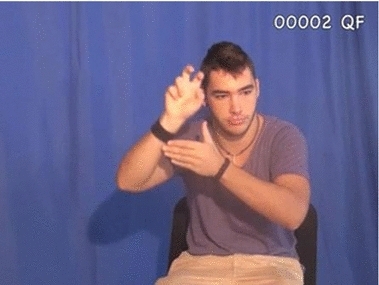
Classifier for ‘legged entity jumping’

Classifiers incorporate two functions: a predicative function and an anaphoric function. For the predicative function one needs to consider the whole classifier construction; that is, not only the handshape but also the movement and the location of the hands. For the anaphoric function, only the handshape is taken into account, because it serves as the link with the antecedent and is the relevant part for the anaphoric chain (Barberà and Quer in press). This anaphoric function is the one crucial for the present proposal, so we have only considered these cases. In order to distinguish them from the purely predicative function, only contexts with classifiers with some intervening linguistic material between the noun and the classifier, as shown in the configuration in (22), have been considered.

**Table:** 

(22)	Noun + CL$_{\mathrm{pred}}$ [...utterance...] + **CL** $_{\mathrm{anaph}}$

Our LSC corpus contains 69 instances of anaphoric strategies. The use of BNs is the strategy most commonly used, which accounts for 55.1% of the cases. The use of entity classifiers is the device that follows, which accounts for 29%, followed by NPs, which represent 11.6% of the data. In the last place, pronouns represent 4.3% of the anaphoric strategies used. These amounts show that pronouns represent the least used strategy. In our analysis for LSC we have considered all four strategies with the goal of offering a broader picture of how anaphoric chains are conveyed in LSC.

In the following section, we analyse the LSC data considering the three main factors that trigger overt marking in spoken Catalan. Each factor is developed and analysed considering the four strategies just presented.

## Results

### Topic Change

In LSC topic change may be achieved with all four anaphoric strategies. The LSC corpus contains 13 instances of topic change, from which 69.2% are expressed with BNs, 15.4% with classifiers, 7.7% with NPs and 7.7% with pronouns. These percentages show that the use of the four strategies is not equivalent, as there is a clear preference for the use of BNs. If we consider the relationship between the kind of strategy used and the possible modality-specific aspects of each strategy (that is, all those aspects tied to the visual-spatial modality, such as the use of signing space or linguistic elements expressed with the different articulators), we observe that in topic change contexts LSC does not rely on modality-specific strategies. In this respect, determiners directed to space and classifiers are very productive strategies typical from the visual-spatial modality, which contribute to building anaphoric chains in signed discourse. On the one hand, both NPs and pronouns rely on the use of signing space for coreferential purposes. Once the antecedent has been introduced and associated with a spatial location, determiners and pronouns directed to that location are understood as coreferential. A pointing sign to a particular location is enough to refer back to the discourse referent. On the other hand, the particular handshape used in classifiers is linked to the antecedent previously introduced and it incorporates rich referential information. Again, the articulation of the classifier is enough to pick up the discourse referent. However, despite the availability of these productive procedures, LSC narratives show a preference for the repetition of the BN in contexts of switch reference. Such an example is shown below.

**Table:** 

(23)	BOY DOG 3-LOOK-3 FROG FISH-TANK 3-LOOK-3 TIME DURATION
	___br ____br
	THEN SLEEP. **FROG** NIGHT ESCAPE CL(3) “legged entity climbs the fish tank and jumps”.
	‘The boy and the dog were looking at the frog in the fish tank for a long time and
	then went to sleep. The **frog**, at night, climbed up the fish tank and escaped.’

The previous context in example (23) is centered on the boy, who is the main topic of the fragment. In the second sentence in (23) the BN for ‘frog’ is enough to change the topic of the first sentence. As the frog was the object in the first sentence, this is an instance of reference to a previous object, which is one of the special cases of topic change as shown also for Catalan in the section ”Topic Change”. The BN refers to the previous object and it is thus used to change the topic of the sentence. Furthermore, the BN is co-articulated with raised eyebrows, as indicated with the horizontal line in the glosses. Eyebrow raise has been considered a characteristic nonmanual marking associated to topic marking (Aarons 1994) but it also fulfils many other functions, such as emphasis, yes/no questions, and it accompanies lexical signs (Kimmelman 2014). In our LSC data, 42% of the instances of topic change where simultaneously expressed with raised eyebrow. Although it does not seem to be an obligatory marking for topic change, it is a quite frequent nonmanual strategy in these contexts. However, given the multiple functions that it fulfils and the restricted genre of discourse considered, we leave for future research the relevance and obligatory nature of eyebrow raise in topic shifts.

The second most frequent strategy in topic change contexts is the use of classifiers, where the hand adopts a handshape that corresponds to some geometrical and visual features of the antecedent. This is a strategy characteristic from the visual-spatial modality. The following example is an instance of a topic change returning to the main topic. The anaphoric strategy, which in this case is expressed with a classifier, serves to close a fragment centered in the bird and to refer back to the previous main topic, which is the boy. Both the classifier for the boy and the classifier for the bird are expressed with the same handshape (8-handshape). However, the movement of the classifier handshape (which fulfils the predicate function), together with the contextual bias are enough to capture the topic change.

**Table:** 

(24)	BOY LOOK DOG RUN CL(8) “legged entity running”. [...] BOY TREE CL(2)
	“two-legged entity climbs a tree and sits in a branch”. IX BIRD FLY CL(8) “bird
	flying” **CL(8) “entity fall down”**.
	‘The boy was looking at the dog, who was running. [...] He climbed up the tree
	and sat on a branch. There, a bird was flying and **he** felt down.

### Focus

Focal information has been found in 8 instances in our LSC data, which are all conveyed with BNs. The basic sign order in LSC is S–O–V (Quer et al. 2005). However, many pragmatic factors may affect this established word order. This is the main reason why in this study word order factors have been left aside and focus instances have been identified depending on the focal particle accompanying the BN. In most of the examples the focal particle is the sign ALSO.

The manual sign ALSO may co-occur with particular nonmanual articulations, which typically consist in raised eyebrows, eyes wide open, and in some cases also head nod. When ALSO co-occurs with the above-mentioned nonmanuals, which are further layered with tensed realisation and head tilt, the scalar additive meaning is obtained and the meaning of ‘even’ is derived (Herrmann 2013). This is shown in the focus example below, where the particular nonmanuals mark a ‘counter to expectations’ meaning.

**Table:** 

(25)	BOY ANIMAL DEER CL(8) “animal running”/CL(2) “two-legged entity on top”
	_ht,br,we
	FAST. ALSO **DOG** CL(8) “animal running” FAST.
	‘The deer was running very fast with the boy lying on his head. Even **the dog** was running very fast.’

### Contrast

Our LSC narratives include 17 instances of contrastive contexts. The most frequent strategy to convey contrast in LSC narratives is the use of BNs (58.8%), followed by pronouns (17.6%), NPs (17.6%) and in the last place, classifiers (5.9%). When we look at the referring expression used in each type of contrast, it is interesting to note that double contrast is mostly expressed with BNs (60% of the cases) and pronouns (40%). In this respect, the use of signing space does not play a role in contexts of double contrast since BNs (which do not include any pointing sign) and pronouns (which are directed to signing space) show a similar behaviour, with the former being slightly more frequent.

In the example of double contrast expressed with pronouns shown below, we find a two-clause discourse in which the subject position is occupied by two different referents (‘we’ and ‘you’) about which opposite actions are predicated, such as ‘go sailing’ and ‘stay’.

**Table:** 

(26)	THEN **IX2.pl** WATER BOAT/SAIL **IX1** STAY.
	‘Now **we** will go sailing in the water and **you** will stay here.’

As for implicit contrast contexts, NPs represent 58.3% of the cases, which differs in great measure with the rest of the referring expressions: BNs represent 25% of the cases, and pronouns and classifiers represent 8.3% each. The great amount of NPs used in implicit contrast shows that the use of space is relevant in conveying implicit contrast. The NP includes a noun followed or preceded by a pointing sign, which implies that another contrasting alternative, which is highly salient in the context, is present although not explicitly expressed. In order for the discourse referent to be contrasted with another entity identifiable in the context, which most likely is spatially established at the opposite lateral area in signing space, BNs alone are not felicitous and signing space is thus very much needed. In the following fragment, the discourse referent ‘family’ is associated with location *a*, as indicated in the glosses, and the boy is associated with location *b*. In the last clause, the implicit contrast is conveyed by the use of the NP ‘the boy’, which includes a pointing localised to the same *b* area, where it was previously associated. This spatial association of the NP evokes an implicit contrast between the boy and the rest of the family, who are associated at the opposed spatial area but not explicitly mentioned.

**Table:** 

(27)	FAMILY IX3.pl$_{\mathrm{a}}$ UPSET SAD IX3$_{\mathrm{b}}$ BOY HAPPY, REASON **IX3** $_{\mathrm{b}}$ **BOY** HUNGRY NOTHING.
	‘The family was upset and the boy was happy because **he** was not feeling hungry at all.’

It should be noted that examples (26) and (27) are very similar to the Catalan examples discussed in (9) and (10): in all these cases pronouns are used to convey either double or implicit contrast. However, such use of pronouns is rare in LSC, where not many cases have been found.

Finally, there are no examples in our LSC sample data instantiating the third type of contrast, weak contrast. In order to study how weak contrast is conveyed in LSC we designed some elicitation tasks and provided the signers with contexts that were expected to trigger a weak contrast context. One of the contexts provided consisted in the same setting found in the *Frog Stories*, where the signer had to visualise herself together with the members of the family at a restaurant. When the waiter would come, she had to play the role of each character. Members had to answer what they wanted without committing themselves about other member’s choice. In the signed contexts obtained, the signers did use pronouns to refer to each member, which were always aligned with particular nonmanual marking. These nonmanuals were articulated on the lower part of the facial expression and they consisted in sucking the cheeks in and pulling the mouth ends down, always combined with a shrug. Therefore, while implicit contrast is conveyed in LSC with pointing signs directed to signing space, weak contrast, and therefore the strongest version of the uncertainty implicature, is captured with nonmanual marking.

Once the three factors that trigger an overt marking in LSC have been presented, it is interesting to note that there are some examples in the data which do not fit in any of the three categories, but rather form a fourth group. They are instances of overt marking in contexts of topic continuation.

### Topic Continuation Factor

LSC behaves as other signed languages, like American Sign Language, which have been analysed as null-subject languages (Lillo-Martin 1986). This means that both agreeing verbs, which inflect for subject and object, and plain verbs, which do not inflect, may omit the subject. The LSC corpus used for this article contains 26 instances of topic continuation with overt marking. Interestingly, the referring strategies used in contexts of topic continuation are restricted to classifiers, BNs and NPs. Pronouns are left aside and not used at all. The most common strategy in topic continuation contexts is the use of classifiers (they account for 76.9% of the cases), which is an expected procedure if we consider the predicative function that classifier constructions have. Since classifiers incorporate the predicate of the sentence, it is expected that this complex construction will be found in anaphoric chains across different sentences. Moreover, previous studies have argued for an analysis of handshape classifiers in terms of gender agreement (Glück and Pfau 1998; Zwitserlood 2003). According to this proposal, the handshape stands as a functional element and functions as an agreement marker, which corresponds to the visual and geometrical properties of the antecedent they refer back to. Therefore, under the analysis of agreement markers, it is very much expected that classifiers may appear in contexts of topic continuation.

However, BNs and NPs (which both account for 11.5% of the cases) are not an expected strategy, as they stand as a repetition of the NP introduced as an antecedent. We hypothesize that this repetitive use of BNs and NPs is genre specific, since it is also attested in narratives directed to deaf children, as well as in narratives directed to adults, such as the Aesop fables (Barberà and Quer in press). Besides the productive strategies typical from the visual-spatial modality, such as the use of space to refer back to discourse referents and the visual-geometrical import of classifiers, in narrative discourse LSC opts for a repetition of BNs and NPs even in contexts of topic continuation, although to a lesser degree compared to classifiers.

As already mentioned, the use of pronouns is not a felicitous strategy in topic continuation contexts. One possible reasoning would be to consider that an overt pronoun always triggers a topic change context, rather than a topic continuation one. In LSC, topic change would be achieved when the direction of the pronoun to signing space is opposed to the previously established one. That is, if for instance the previously introduced antecedent is established in the ispsilateral area (the signing space area close to the dominant hand, which is the right hand for right-handed signers) and the overt pronoun in subsequent discourse is directed to the contralateral area (the area close to the non-dominant hand), a topic change context would arise. Therefore, the overt pronoun together with the corresponding spatial location associated is a strategy not found in topic continuation contexts, but it is more productive in topic change ones.

## Discussion and Conclusions

This article has presented an examination of the uses of overt subject pronouns in Catalan and how anaphoric contexts are rendered in a language that exploits the visual-spatial modality, such as LSC. A correspondence in the classification of functions of anaphoric strategies that the two languages use has been shown, taking into account the different instantiations that serve a reference-tracking function.

One of the main findings of the comparison concerns the use of pronouns in LSC. We were expecting to find pronouns in LSC in the contexts in which pronouns are used in Catalan: that is, topic change, focus and contrast. However, we found that the frequency of pronouns in LSC is very low. This is particularly surprising given the fact that sign language pronouns are signs directed towards a particular area in signing space and this use of space is a very productive strategy: once the discourse referent is introduced into the discourse, and therefore associated with a spatial location, a pointing sign directed to it should be enough to refer back to it. However, the use of pronouns is the least used strategy. A possible explanation for this is that the particular discourse genre licenses the use of anaphoric strategies that are informatively richer.

Another surprising result is that the LSC data has shown an important preference for the use of BNs in both topic change and focus contexts. Again the results for LSC in these contexts show a preference for strategies that are not tied to the visual-spatial modality. The great use of BNs could be considered a non-expected strategy in cohesive narrative discourse. However, we have argued that this repetition may be due to a genre-specific strategy.

An important distinction has been found in contexts of contrastive meaning, and more concretely when dealing with the three kinds of contrast. BNs are the primary means to convey double contrast. Implicit contrast relies in the use of pointing signs directed to space co-articulated with the noun. The use of signing space, as a modality-specific feature characteristic of sign languages, evokes a contrastive set of alternatives that are not explicitly expressed. Finally, uncertainty in weak contrast contexts is conveyed with particular nonmanual marking articulated in the lower part of the facial expression.

As the first comparison study between overt anaphoric strategies in two languages of different modality, some issues have not been included in this article. On the one hand, role shift structures, a mechanism typical from signed languages where the signer adopts the role of a character of the story, have not been taken into account. Role shift is a way of maintaining an active topic and it is mainly expressed with change in the nonmanuals. In its quotational use, it is used to directly report the speech or the thoughts of a character. This strategy would then need to be compared with spoken Catalan intonation to represent the speech of the character and has been left for future research. Another strategy that has not been treated here is simultaneous constructions; that is the use of the dominant and the non-dominant hand to express two actions that are happening simultaneously. This strategy allows preserving a topical sign across several sentences (mainly expressed with the non-dominant hand) and may affect the use of different referring expressions. Forthcoming research should take into account simultaneous constructions.

Finally, another issue we have left for future research is the study of the role that rhetorical relations have in licensing a particular anaphoric expression. Kehler (2002) shows how the interpretation of pronouns is heavily influenced by the type of rhetorical relation marked in the segment. A significant part of the unaccounted data for Catalan showed a particular rhetorical relation and it is an open question whether this could be used to analyze these problematic examples or even a greater amount of data.

## Footnotes

1
http://nocando.barcelonamedia.org/.

2
http://blogs.iec.cat/lsc/corpus/.

3 The following abbreviations are used in the glosses of the Catalan examples: 3SG (3rd person singular), 2SG (2nd person singular), AUX.PST (past tense auxiliary).

4 We omit the glosses in the longer corpus examples for the sake of clarity. A null pronoun in the original will be marked in the literal translation as [null]. The intended referent will be signaled by means of a subscript after the null or overt pronoun.

5 We follow the usual glossing conventions in the sign language literature, according to which manual signs are represented by the capitalized word corresponding to the translation of the sign. The relevant abbreviations for the purposes of this article are the following: IX# (pointing sign; the numbers refer to the grammatical person); #-VERB-# (verb agreeing with subject and object); CL (classifier construction). In a classifier construction, the handshape is indicated in parentheses, followed by a rough meaning description. The handshape is typically indicated with the number that corresponds to a hand configuration when articulating that particular number. A horizontal line above the glosses indicates the scope of nonmanuals: br (eyebrow raise), hd (head tilt), we (eyes widened).
